# Design and methods for a cluster-controlled trial conducted at sixty-eight daycare facilities evaluating the impact of “JolinchenKids – Fit and Healthy in Daycare”, a program for health promotion in 3- to 6-year-old children

**DOI:** 10.1186/s12889-017-4551-x

**Published:** 2017-07-11

**Authors:** Berit Steenbock, Hajo Zeeb, Stefan Rach, Hermann Pohlabeln, Claudia R. Pischke

**Affiliations:** 1 Leibniz Institute for Prevention Research and Epidemiology – BIPS, Bremen, Germany; 2 Health Sciences Bremen, University of Bremen, Bremen, Germany

**Keywords:** Health promotion program, Daycare, Preschoolers, Body composition, Motor skills, Physical activity, Accelerometry, Eating habits, Mental well-being

## Abstract

**Background:**

The prevention of overweight and obesity during infancy is a highly relevant public health topic given the significant impact of childhood obesity on acute and chronic diseases, general health, and well-being in later stages of life. Apart from the family setting, daycare facilities (DFs) represent a key setting for health promotion among children under the age of six years. “JolinchenKids – Fit and Healthy in Daycare” is a multi-component program promoting physical activity (PA), healthy eating, and mental well-being in 3- to 6-year-old preschoolers at DFs, originally designed by the German health insurance AOK. To evaluate program effectiveness, a cluster-controlled trial involving 68 DFs is currently conducted. The objective of this article is to describe the background, study design, and aims of this trial.

**Methods/design:**

Sixty-eight DFs across Germany will be recruited to take part in the study, half of them serving as intervention DFs and half of them as delayed intervention control DFs (which receive the program upon completion of the study). At each DF, height, weight, and body composition, as well as motor skills, will be assessed in twenty 3- to 6-year-old children. Children’s eating and PA habits, and mental well-being will be assessed via parental questionnaires. A subsample of children (i.e., at 24 DFs which are randomly selected within a geographic region) will be asked to wear accelerometers at their wrists to objectively measure PA over the course of seven days. To compare changes in body composition, motor skills, eating and PA habits, and mental well-being of children at intervention DFs with those observed among children at delayed intervention control DFs over one year, all measurements will take place at baseline and twelve months after the launch of the program at all DFs.

**Discussion:**

This study investigates the influence of a health promotion program in the daycare setting on various outcomes, including body composition and objectively measured PA, in a nationwide sample of 3- to 6-year-old preschoolers. This study will provide evidence regarding the effectiveness of a multi-component program for health promotion in this setting and may provide insights into new strategies for preventing obesity in early life.

**Trial registration:**

German Clinical Trials Register DRKS00011065 (Date of registration 16–09-2016).

## Background

Data from the German Health Interview and Examination Survey for Children and Adolescents (KiGGS) indicate that one in eight children under the age of six is either overweight or obese in Germany [1]. Childhood overweight and obesity is accompanied by multiple negative health consequences in the short and long term [2–6]. There is also clear evidence that excessive weight gain in the first years of life increases the risk for obesity and chronic diseases, such as coronary heart disease, later in life [7–9]. In fact, obese preschool-aged children are five times more likely to be overweight during adolescence and have a four times increased risk to be obese in adulthood when compared to normal weight children [10]. Efforts to date have failed to halt the rise in childhood obesity. The implementation of comprehensive programs that promote the intake of healthy foods and physical activity (PA) and aim to reduce sedentary behavior in children and adolescents is therefore strongly recommended [11].

Unhealthy eating and PA habits which clearly contribute to the etiology and progression of obesity, are, to a great extent, learned during the first years of life and tend to persist into adulthood [12, 13]. Therefore, this period in life represents a powerful opportunity for impacting energy balance-related behaviors. In Germany, to date, 50% of children under the age of six years do not meet the current PA recommendation for their age group [14] and only one fifth of 3- to 6-year-old children consume the recommended five or more portions of fruits and vegetables per day [15]. To date, even well-designed studies examining the effects of interventions targeting these two behaviors to prevent or reduce childhood overweight and/or obesity yielded no, inconsistent, or only weak effects [16]. To conclude, there is still a lack of studies demonstrating substantial or clinically meaningful changes in body composition and eating and PA habits under real-life conditions in this population.

Daycare facilities (DFs) are a key setting for early health promotion. Findings of a recent systematic review suggest that there is a strong potential to shape health-relevant attitudes and behaviors in the daycare setting [17] and some research indicates that parents may easily be involved in daycare-based interventions [18]. Furthermore, many children spend the majority of their waking hours in this setting. For example, in Germany, more than 3.5 million children are taken care of in DFs, 27% for more than seven hours every day [19].

“JolinchenKids – Fit and Healthy in Daycare” is a multi-component program, originally designed by the German health insurance AOK, aimed at modifying the daycare environment and individual health behavior among children. Both, daycare staff and parents, are involved in the implementation of the program. Because data gathered in qualitative research in the school setting suggest that administrators and teachers feel under-qualified to deliver nutritional information [20], training in delivering the intervention and intervention messages is offered to daycare staff by external specialists in the fields of nutrition, PA, and mental well-being over a three-year period as part of the program.

In the following sections, the research design and methods of a cluster-controlled trial (CCT) evaluating the impact of “JolinchenKids – Fit and Healthy in Daycare” at 68 daycare facilities are described. The main objective of this trial, funded by the German health insurance AOK, is to evaluate program-associated changes in body composition, behavioral and psychological outcomes in a nationwide sample of preschoolers comparing children enrolled at 34 intervention DFs with those enrolled at 34 delayed intervention control DFs.

## Methods

### Study aims

The primary aim of the study is to evaluate whether children participating in the program at the 34 intervention DFs demonstrate greater changes in body composition and behavioral and psychological outcomes compared to those enrolled at the 34 delayed intervention control DFs. Secondary aims of the study are:

(I) To evaluate whether the daycare environment at DFs is modified as part of the program to support changes in individual health behavior of children;
(II) To examine whether DF staff make lifestyle changes recommended to them as part of the program;
(III) To adapt future program implementation based on results gathered in focus groups with DF staff, parents, and preschool-children on factors facilitating or hindering implementation.

### Study design

Due to the risk of cross-contamination of intervention effects, it was not feasible to allocate children to the intervention or non-intervention conditions at individual DFs [21]. Randomization of DFs to intervention vs. control conditions was also not possible (see below for reasons and recruitment of DFs in the study). Therefore, this study was planned as a CCT with a non-random allocation procedure. CCT designs are used not only to evaluate group interventions but also individual interventions where group level effects are relevant [22]. Therefore, clusters in this study constitute the kindergarten groups of the individual DFs (one group per DF). These clusters will be allocated to intervention and control groups and outcomes will be measured among individuals within those clusters.

### Inclusion criteria

DFs have to fulfil the following criteria to be included in the study: (1) > 14 children aged three to five years are currently taken care of at the individual DF; (2) the DF is organized in groups; (3) there is sufficient room for conducting the tests for assessing motor skills; and (4) the head of the DF agrees to participate in the study and signs a letter of consent. DFs will be excluded if they do not meet these criteria.

### Participants and procedures

Ethical approval for the study was obtained from the Medical Association in Bremen (HR/RE – 522). For the recruitment of 68 DFs, the following recruitment strategy will be employed. The health insurance AOK determines when DFs start with the implementation of the intervention. Therefore, it is not feasible to randomly allocate DFs to either intervention or delayed intervention control conditions. To facilitate the recruitment process, the AOK will provide two lists, each of them containing addresses of possible intervention or delayed intervention control DFs from various German federal states. In each federal state, DFs from both lists will be randomly selected and invited to participate in the study via mail. Heads of DFs will receive general information on the study and a short questionnaire. In this questionnaire, the following data will be assessed to later match intervention and delayed intervention control DFs: Estimates of the percentage of parents with lower levels of education and income at the DF, estimated percentage of parents with migration background, and other indicators for the distribution of socio-economic status among parents (i.e., the percentage of parents receiving subsidies from the government for payment of DF fees, percentage of single parents). In addition, this questionnaire will include questions that help the research staff organize baseline and follow-up assessments (e.g., number of children between the ages of three and five years at the DF). The heads of DFs of the DFs fulfilling the inclusion criteria described above will be asked for a written consent to participate in the study and will subsequently be contacted via telephone to arrange the dates for baseline and follow-up assessments. Heads of DFs will subsequently be in charge of the recruitment of the children at their facility. We expect that the heads of DFs will be familiar with all parents which may facilitate recruitment of children. Parents and their children will then be recruited to the study via written invitations which are provided to the heads of DFs by the research institute and forwarded to the parents by them. In addition, a telephone hotline will be made available to parents to answer questions arising during recruitment. A maximum of 25 children per DF will be invited to participate in the study.

Parents will be informed that they and their children can withdraw from the study at any time. To participate in the study, children have to agree to participate and parents will be asked to give their written consent and to complete the parental baseline and follow-up questionnaires. Heads of DFs or other DF staff assigned to recruitment will collect the completed written consent forms and questionnaires from the parents (at baseline and follow-up) and will send them to the research institute in the mail.

### Sample size

Sample size was calculated with regard to the two primary outcomes diet and PA. Based on a prevalence of fruit consumption reported in a previous study conducted at 64 German DFs including 2.658 preschoolers which reported rates of fruit consumption of 66.7% in the intervention group and 56% in the control group [23], we assumed a difference in the change in diet from baseline to 12-months follow-up of 8% between intervention and control group considering an estimated intracluster correlation coefficient (ICC) of 0.02, 90% power, and α = 0.05 (two-sided). Based on this, we estimated that a total of 1.020 children at 68 DFs would be required for our study. With regard to more conservative estimates for the ICC, this sample size will provide more than 80% power to detect changes of 8% in fruit consumption. We assumed a 25% loss to follow-up twelve months post-baseline. Therefore, we aim to reach 1360 children, in total, at baseline, twenty children at each of the 68 participating DFs. According to De Bock et al. [24], little is known about the extent to which PA-related outcomes in preschool children are inter-correlated. Therefore, our sample size calculation for detecting changes in objectively measured PA is based on the conservative assumption of an ICC of 0.1. To detect a difference of 0.5 standard deviations between intervention and control groups, we assumed a standard deviation of 38 counts per 15 s [24], a power of 90%, and α = 0.05 (two-sided), and estimated that a total of 360 children at 24 DFs will be necessary for our study. We increased our recruitment goal to 20 children per DF (240 per arm) to account for a 25% loss to follow-up. All power calculations were carried out in PASS [25], using tests for two proportions in a cluster-randomized design function.

### Assessments

Children in each DF will be examined and tested during the same day. Anthropometric measures and motor tests will be performed in the gyms of the DFs between 8 a.m. and 4 p.m. during the day of the assessment. Children will not wear shoes during the weight and height measurements. To accomplish assessments, two study nurses will travel separately from DF to DF throughout Germany.

A portable stadiometer (Seca®, type 213) will be used to measure height and a body composition analyzer (TANITA®, BC 420 SMA) to measure weight and body composition (i.e., body-mass index, percent body fat). After the anthropometric measurements, children will participate in the whole battery of the KiMo test, a motor screening for preschool children aged three to six years consisting of five test items, each of them covering one motor ability relevant at the preschool age. A detailed description of the testing procedure is given by Klein and colleagues [26]. In summary, the tasks of this test (and the corresponding motor abilities in parentheses) are the following: 1) shuttle run (speed), 2) standing long jump (speed strength), 3) one leg stand (coordination, static balance), 4) sit and reach (flexibility), and 5) lateral jumping (coordination, strength endurance). Age- and gender-specific reference values are available for 3- to 6-year-old children. Test-retest values of the test demonstrated reliability and objectivity. Correlation coefficients amounted to *r* = .8 for the shuttle run, the standing long jump, lateral jumping, and the sit and reach; and a moderate correlation coefficient of *r* = 0.6 was found for the one leg stand [26]. Study nurses will be trained in administering this test prior to its use in the field.

In a subsample of children at 24 DFs (12 intervention and 12 delayed intervention control DFs, ca. *n* = 360), PA will be assessed objectively using wrist-worn triaxial accelerometer watches (GENEActives, produced by ActivInsights Ltd.) for seven consecutive days at baseline and follow-up. The GENEActives provide raw accelerometry data in three axes (activity counts measured at 100 Hz) and measure surface skin temperature. They were chosen for the study because they are light-weight and waterproof and can be worn at the wrists meaning that they are more comfortable for continuous 24 h use among small children compared to the hip-worn version of the activity monitor. Compliance among small children has been shown to be greater when using wrist-worn accelerometers compared to hip-worn devices [27]. Reliability and validity of GENEActive measurements in children have been widely demonstrated for children above the age of eight years [28, 29]. The validity of this activity monitor for preschool-aged children is currently examined in another study [30]. Study nurses will mount the device on the left wrist because the majority of children is expected to be right-handed and it is recommended to use the non-dominant hand for measurement because energy prediction is more precise this way [29]. Handedness will also be assessed via parental questionnaire and controlled for in later statistical analysis. Parents of children of this subsample receive instructions regarding the recommended wear time and properties of the device, and a paper-and-pencil diary to report wear and non-wear times and to document reasons for not wearing the device.

The parental questionnaire will include parent-reported height and weight of the children, as well as self-reported height and weight of the parents, and items assessing behavioral and socio-demographic characteristics of both, children and parents. To assess the socioeconomic status of families included in the study, information on education and professional status of parents, as well as total income available to the family household, will be collected. Questions regarding migration background will include nationality, country of birth, year of immigration of both parents, and languages spoken at home. For information about the medical history of children, a selected number of physician-diagnosed chronic conditions (e.g., hayfever, atopic dermatitis, and asthma) will be included in the questionnaire, as well as items for assessing days of sickness and doctor’s visits during the past 12 months. Selected socio-demographic characteristics and health behaviors are also assessed in DF staff. Baseline and follow-up questionnaires employed at intervention and delayed intervention control DFs will be identical (for further detail, see Table 1).

**Table 1:** Overview of the content of the questionnaires employed in the study

Assessment method	Measure of interest	Variables	Instrument/Scale
PARENTAL QUESTIONNAIRE			
Characteristics of parents	Socio-demographic characteristics	Age, education, employment, net household income, migration background	KiGGS [34]^a^
	Health	Height, weight, self-rated health status	SF-12, only one item [35]
	Smoking	Current smoking, intention to quit	HAPA, intention [36]
	PA	PA and sports activities during past three months, intention to engage in PA	DEGS [37]^b^, HAPA, intention [36]
	Diet	Intention to consume fruits and vegetables	HAPA, intention [36]
	Family climate	Health behaviors performed and health information available in the family context	FHC-scale [38]
Characteristics of children	Socio-demographic characteristics	Age, sex	
	Health	Height, weight, days of sickness, parent-rated health status, physician-diagnosed chronic conditions, doctor’s visits	SF-12 [35], KiGGS [34]^a^
	Diet	Consumption of un-sweetened beverages, fruits and vegetables, milk products, snacks, number of meals per day	Self-developed quantitative FFQ
	PA	Hours per week of moderate to intense PA and sports club membership	IDEFICS [39]^c^
	Media consumption	Time spent watching TV, video, DVD or using the computer, tablet, smartphone or game console	IDEFICS [39]^c^
	Mental well-being	Health-related quality of life, psychological attributes	Kiddy-KINDL^R^ [40], SDQ [41]
DF STAFF QUESTIONNAIRE			
Characteristics of DF staff	Socio-demographic characteristics	Age, sex, employment, migration background	DEGS [37]^b^
	PA	Work activity, sports activity, leisure-time activity; intention to engage in PA	Baecke questionnaire [42], HAPA, intention [36]
	Diet	Consumption of un-sweetened beverages, fruits and vegetables, milk products, snacks, number of meals per day; intention to consume fruits and vegetables	Self-developed quantitative FFQ, HAPA, intention [36]
	Smoking	Current smoking, intention to quit	HAPA, intention [36]
	Health	Self-rated health status, health-related quality of life, use of healthcare services, preventive health check-up program, preventive services (past 12 months)	SF-12 [35], DEGS [37]^b^
	Self-efficacy	General self-efficacy	GSE [43]
	Mental well-being	Stress	PSS4 [44]
	Health literacy	Knowledge, motivation and competences to access, understand, appraise, and apply health information	HLS-EU-Q47 [45]

Abbreviations: *DEGS* German Health Interview and Examination Survey for Adults, *FFQ* Food Frequency Questionnaire, *FHC* Family Health Climate, *GSE* General Self-Efficacy, *HAPA* Health Action Process Approach, *HLS-EU* European Health Literacy, *KiGGS* German Health Interview and Examination Survey for Children and Adolescents, *IDEFICS* Identification and Prevention of Dietary and Lifestyle induced Health Effects in Children and Infants, *KINDL* ^R^ revised questionnaire to assess Health-Related Quality of Life in children and adolescents, *PA* Physical activity, *PSS4* Perceived Stress Scale, *SF-12* Short Form-12, *SDQ* Strengths and Difficulties Questionnaire

^a^Items regarding socio-demographic characteristics of parents will be taken from the parental questionnaire for 3–6 year-olds from the KiGGS

^b^Items regarding parental PA and DF staff’s health and socio-demographic characteristics will be taken from the health questionnaire for 18–64 year-olds of the DEGS

^c^Items regarding children’s PA and media consumption will be taken from the parental questionnaire of the IDEFICS study

Six months after the launch of the program, computer-assisted telephone interviews (duration: ca. 20 min) will be conducted with DF staff in charge of implementation at all intervention DFs. Changes in institutional structures and processes that may facilitate the implementation of the program (e.g., provision of un-sweetened beverages at a regular basis, creation of space in the DFs enabling children to engage in active play [outside areas, gyms]) will be assessed.

### Follow-up assessment and delayed intervention

The follow-up assessment will take place approximately twelve months post-baseline and the same tests and questionnaires will be employed as those employed during baseline assessment. After completion of the follow-up assessment, the program will be made available to the delayed intervention control DFs. However, program implementation and changes in the outcomes which were assessed at the intervention DFs will not be further tracked at delayed intervention control DFs.

### Intervention

The three-year program ‘JolinchenKids - Fit and Healthy in Daycare’ was originally designed by the German health insurance AOK (see https://bremen.aok.de/inhalt/jolinchenkids-2/ for further detail). It is comprised of five modules, three focusing on children, one on parental participation in the program, and one on promoting health among DF staff. DFs are free to choose which modules they would like to implement and in which order meaning that there is no fixed time schedule to follow in regard to the implementation of these modules. The three modules focusing on children’s health are designed to affect dietary and PA habits among 3- to 6-year-olds and to improve their mental well-being. Instructions for intervention implementation are outlined in a detailed manual and DF staff receives training in one two-day workshop before the start of the program.

Activities of the *diet module* include small changes in the DF environment. For example, un-sweetened beverages, such as tea and water, are made available throughout the day in the groups (“drinking oasis”) and a healthy breakfast buffet is offered once every week (in existing or newly created areas in those DFs that did not have eating areas before). Furthermore, children are offered raw foods (e.g., fruits) twice every day. Examples for program activities in the *PA module* include the provision of a box with cards for kindergarten teachers with instructions for PA games with children. Also, intervention efforts are put into providing or creating areas with sufficient space at DFs so that children can involve in active play. The program recommendation for PA is at least one hour per week of engagement in PA games. The module focusing on *mental well-being* includes, among other materials, the provision of a card box with techniques for stress management to be implemented by DF staff during the day (program recommendation: one hour per week). Further, a space in the group room is created that children can take breaks in when they need them. The module focusing on *parental participation* in program activities includes several actions to encourage parents to support program implementation, such as parental participation in small implementation teams at DFs that plan implementation at the individual facility. DF staff is trained in how to involve parents in the program during a one-day workshop. In addition, materials for health education are distributed to parents in the form of newsletters (once per year per intervention module) or so called “messages in a bottle” (i.e., take home instructions for PA games or recipes for healthy meals). The fifth module is focused on *health promotion among DF staff*. DF staff is provided with a brochure containing information regarding health behavior change and the incorporation of health behavior in day-to-day activities at the DF. Furthermore, staff at the DFs is offered one educational workshop entitled “Fit at the job” lead by employees of the health insurance during which information on how to live a healthier lifestyle is provided to staff. Furthermore, counseling and classes (e.g., progressive muscle relaxation, pilates, nordic walking) are offered to DF staff by the health insurance at a regular basis. Table 2 provides further detail on the intervention components.

**Table 2:** Overview of the intervention components of ‘JolinchenKids - Fit and Healthy in Daycare’

Module “Diet”	
(1) DF environment	• “drinking oasis” and “colorful garden” (un-sweetened beverages throughout the day, raw foods twice per day)
(2) Wooden train	• consisting of six wagons, each of them representing a food group; to be used by DF staff to teach children about food groups
(3) Card box “Healthy-and-tasty-land”	• one activity/ game per week (e.g., excursion, recipe, quiz)
(4) Healthy breakfast buffet	• once per week
(5) Joint activities with parents	• twice per year (e.g., sugar quiz)
(6) Newsletters	• e.g., instructions for healthy recipes distributed to parents once per year
Module “Physical Activity”	
(1) DF environment	• creating areas with sufficient space for active play
(2) Card box “Get-fit-in-the-jungle”	• at least one hour per week of engagement in PA games to enhance static balance, flexibility, strength, endurance, and speed
(3) Joint activities with parents	• twice per year (e.g., “family olympics”)
(4) Newsletters	• e.g., general information about PA, ideas for PA games to be included in everyday family life distributed to parents once per year
Module “Mental Well-being”	
(1) DF environment	• creation of a space where children can take breaks
(2) Card box “Feel-good-island”	• use of the hand puppet named “Jolinchen”
	• one hour per week (e.g., handling of conflicts, identifying and describing emotions)
	• short timeouts when needed (e.g., employment of techniques for stress management)
(3) Workshop for parents entitled “How to nurture children’s wellbeing”	• once per year executed by DF staff
(4) Joint activities with parents	• twice per year
(5) Newsletters	• information about card box topics distributed to parents once per year (e.g., identifying and describing emotions, handling of conflicts, stress management, strengthening of self-image of children)
Module “Parental Participation”	
(1) “JolinchenKids” team	• establishment of a team to organize implementation (DF administrator, DF staff, parents)
(2) Workshop “How to engage parents” for DF staff	• executed once during the 3-year implementation period by employees of the health insurance
(3) Manual “Parents are joining”	• practical information for DF staff about how to engage parents in program activities
(4) Card box “Joint activities with parents”	• general information about joint activities with parents
(5) Newsletters	• module-specific information and suggestions regarding games to be integrated into day-to-day family life distributed to parents once per year per module
(6) “Message in a bottle”	• take home instructions for PA games or recipes for healthy meals to be distributed to each child for a time period of two weeks
Module “Health Promotion among DF staff”	
(1) Workshop “Fit at the job”	• once in three years executed by employees of the health insurance
(2) Manual “How to live healthier”	• information regarding health behavior change and the incorporation of health behavior in day-to-day DF activities
(3) Further counseling and classes	• preventive healthcare services provided by the health insurance (e.g., classes in progressive muscle relaxation, pilates, nordic walking)

### Tracking implementation progress at intervention DFs

Conclusions drawn from the data gathered in this trial will highly depend on the module choices at the individual DFs, as well as on the amount of time intervention DFs spend on implementing program activities. To assess intervention dose and fidelity, intervention DFs will therefore be provided with paper-and-pencil calendars to track implementation progress at individual DFs over a period of 12 months. Using these calendars, DF staff will document module choices, as well as module-specific environmental changes and activities at intervention DFs every week. To foster compliance, the calendar is kept simple and easy to use. Directly after the baseline assessment, intervention DF groups receive the calendars including calendar pages for 12 months. Every three months, DF staff will be prompted to send the completed calendar pages back to the research team via mail. The calendar covers program-associated activities from the modules “*diet*”, “*physical activity*”, “*mental well-being*” and “*parental participation*”. For each module, intervention components are listed (e.g., “drinking oasis”, “colorful garden” healthy breakfast buffet, card boxes). The DF staff will be asked to mark the checkboxes, as well as to document the weekly amount of time (in minutes) spent on working with certain intervention materials (e.g., card boxes, wooden train).

#### Qualitative research to gather information about facilitating and hindering factors regarding program implementation at intervention DFs

Four focus group discussions with DF staff, parents, and children will be held at intervention DFs throughout Germany to gather information about factors facilitating or hindering program implementation and general program acceptance. Participating DFs will be randomly selected per geographic region. Attendance of five to ten persons will be intended per focus group discussion at each DF. Focus group discussions will be led by a researcher of the project team, using an adapted focus group guide from a previous study [31]. Researchers leading the focus groups will obtain an informed consent from participants.

### Analysis strategy

#### Quantitative analysis

To evaluate intervention effectiveness, changes in body composition, health behaviors, and psychological outcomes from pre- to post-test among children at intervention DFs will be compared to those observed in children enrolled at delayed intervention control DFs. In addition to descriptive data analyses, quantitative analyses will be performed involving simple two-sample t-tests, as well as mixed effects regression models. Such regression models account for the clustered structure of the data, allow for controlling of baseline differences between the groups and for considering potential confounders, such as age, gender, or parents’ educational attainment, simultaneously. All statistical analyses will be carried out using SAS 9.3 software (SAS Institute Inc., Cary, NC) [32].

#### Qualitative analysis

Qualitative focus group discussions will be protocolled by one researcher and audio-recorded. The audio-records will be transcribed, using the software f4. Deductive methods will be used for analyzing the data. Based on the interview guide, an analysis matrix with different categories will be developed. The content of the final protocols will be transferred to each category of the analysis matrix.

### External advisory board

An advisory board of national researchers with expertise in the promotion of healthy dietary patterns, PA, and mental well-being in children will provide advice regarding the development of the assessment battery and the focus group guide. After that, the research team will provide regular status updates on the project to the advisory board (at least three times during the funding period).

### Expected results

We expect that children participating in the program demonstrate greater improvements in body composition, motor skills, dietary and PA patterns, and mental well-being over the course of 12 months than those not participating in the program. For example, we expect greater increases in the scores of the KiMo test, fruits and vegetable consumption, time spent on moderate to vigorous PA, and health-related quality of life, and more pronounced reductions in sitting time and un-sweetened beverage consumption among children participating compared to those not participating in the program.

## Discussion

The provision of evidence-based multi-component interventions to young children under “real-world” conditions which promote healthy eating, PA, and mental well-being is currently very limited in Germany. Thus far, only few multi-component interventions have been implemented and scientifically evaluated at German DFs. Also, implementation research investigating implementation processes, as well as individual or environmental factors facilitating implementation of such interventions, is still lacking [33]. Results of this study will provide new information regarding the effectiveness of a health insurance developed multi-component intervention which targets children’s individual health behavior and changes in the daycare environment. We hope to obtain new insights into the implementation and effects of this program for researchers and stakeholders in the preschool setting and to derive recommendations for the uptake and implementation of such programs in the future.

## Abbreviations

CCT: Cluster-controlled trial
DEGS: German Health Interview and Examination Survey for Adults
DF: Daycare facility
GSE: General self-efficacy
HAPA: Health Action Process Approach
HLS-EU: European Health Literacy
ICC: Intracluster correlation coefficient
IDEFICS: Identification and Prevention of Dietary and Lifestyle-induced Health Effects in Children and Infants
KIGGS: German Health Interview and Examination Survey for Children and Adolescents
PA: Physical activity
PSS: Perceived Stress Scale
SDQ: Strengths and Difficulties Questionnaire
SF: Short Form
WHO: World Health Organization
